# Viral hepatitis C – a challenge for the public health

**DOI:** 10.1093/eurpub/ckac131.533

**Published:** 2022-10-25

**Authors:** I Pakov, K Terzieva, M Kunchev, D Shalamanov, M Karcheva, G Gancheva

**Affiliations:** 1 Department of Infectious Diseases, Epidemiology, Parasitology and Tropical Medicine, Medical University-Pleven, Pleven, Bulgaria; 2 Laboratory of Virology, Military Medical Academy, Sofia, Bulgaria; 3 Scientific Centre of Military Epidemiology and Hygiene, Military Medical Academy, Sofia, Bulgaria

## Abstract

**Background:**

Viral hepatitis C (VH C) is a global health problem with overall prevalence in 3-5% of the human population. This study provides the relevance of viral genome characterization in clinical settings.

**Methods:**

Retrospective study was conducted upon epidemiological, demographic, clinical, laboratory and viral characteristics in fifty cases of VH C confirmed with positive anti-HCV, evaluated by ELISA. Thirty eight of cases were hospitalized in different clinics of the University Hospital “Dr Georgi Stranski” - Pleven (2017-2018) and remainders were blood-donors registered in Regional Center of Transfusion Hematology - Pleven. The viral load and genotype of HCV had been investigated by Real-Time PCR in Laboratory of Virology at Military Medical Academy - Sofia.

**Results:**

The prevalence of cases was equal and highest in age groups 30-39 years and 60-69 years (24%, respectively). Males were 69.81% (p < 0.05). Surgical interventions (26.32%), blood infusions (23.68%) and hemodialysis (15.79%) were at highest risk for VHC (p > 0.05). Thirty hospital patients were with chronic VH C (78.95%) (p < 0.05). Clinical symptoms suggestive viral hepatitis were adynamia (39.47%; OR 5.25), anorexia (28.95%; OR 2.16), heaviness in the abdomen (21.05%; OR 23.33), and 52.63% of patients were asymptomatic (p < 0.0005). Laboratory investigations revealed slightly or moderately elevated total bilirubin (mean 53.27±37.38 µmol/L; 95% CI 18.48-88.06) and transaminases - ASAT (mean 231.36±155.82 IU/L; 95% CI 79.91-382.80) and ALAT (mean 294.48±196.26 IU/L; 95% CI 96.37-492.59) (p > 0.05). Investigation of viral load of HCV revealed 22 samples with detectable viral load (range 683-673 720 copies/ml). All isolates of HCV had been proved to be genotype 1b.

**Conclusions:**

VH C is mostly asymptomatic. Screening for anti-HCV in risk groups and genotyping of HCV will improve surveillance, reduce nosocomial HCV-infections, facilitate therapeutic management and prevent complications of infected individuals.

**Key messages:**

• Screening for anti-HCV in risk groups and genotyping of HCV improves surveillance and reduces nosocomial HCV-infections.

• Screening for anti-HCV facilitates therapeutic management and prevents complications of infected individuals.

